# The Influence of the Tissue Adhesive Material as a Surgical Wound-Closure Technique Following Carpal Tunnel Decompression on Neurological and Functional Outcomes: A Single-Center Randomized Controlled Trial

**DOI:** 10.7759/cureus.53312

**Published:** 2024-01-31

**Authors:** Veridijana Sunjic Roguljic, Luka Roguljic, Vedran Kovacic, Ivica Bilic, Ivana Jukic

**Affiliations:** 1 Department of Surgery, Division of Plastic, Reconstructive and Aesthetic Surgery With Burn Care, University Hospital of Split, Split, HRV; 2 Department of Surgery, Division of Orthopaedics and Traumatology, University Hospital of Split, Split, HRV; 3 Department of Internal Medicine, Division of Emergency and Intensive Medicine With Clinical Pharmacology and Toxicology, University Hospital of Split, Split, HRV; 4 Department of Neurology, Division of Clinical Neurology, University Hospital of Split, Split, HRV; 5 Department of Internal Medicine, University Hospital of Split, Split, HRV

**Keywords:** hand function, cyanoacrylate, skin suture, skin adhesive, carpal tunnel

## Abstract

Background

Carpal tunnel syndrome (CTS) is caused by compression of the median nerve in the carpal tunnel. The effect of tissue adhesives as a material for wound closure following CTS decompression has been insufficiently investigated. This study aimed to evaluate outcomes by comparing two modalities of wound closure following carpal surgery in patients randomly assigned to either tissue adhesives or sutures.

Methodology

This randomized, prospective study was conducted in April 2022 at the University Hospital of Split in Croatia. Patients aged 61.56 ± 12.03 years were randomized to either tissue adhesive Glubran Tiss 2®-based (n = 50) or suture-based (n = 50) wound-closure techniques. The following outcomes were assessed before surgery and six months postoperatively: hand strength, electroneurographic characteristics of the median nerve, and the Boston Carpal Tunnel Questionnaire.

Results

Significant differences between glue-based and suture-based wound-closure techniques were found in the six-month postoperative hand grip strength (25.06 ± 6.69 vs. 21.41 ± 5.62 kg; p = 0.002), postoperative sensory amplitude (10.08 ± 5.50 vs. 7.54 ± 5.41 mV; p = 0.012), and postoperative sensory velocity (42.22 ± 11.04 vs. 35.23 ± 16.40 m/s; p = 0.008). In the glue-based group, significantly more patients achieved a postoperative sensory velocity greater than 45 m/s (47.9% vs. 22.0%; p= 0.006), postoperative distal sensory latency less than 3.5 ms (89.6% vs. 84.0%; p = 0.304), and postoperative motor latency of less than 4.2 ms (60.42% vs. 38.00%; p = 0.022).

Conclusions

This trial demonstrated that cyanoacrylate-based adhesion material for wound closure after open CTS decompression compared with sutures showed a significant six-month postoperative increment in hand grip strength and median nerve sensory conduction.

## Introduction

Carpal tunnel syndrome (CTS) is the most prevalent peripheral neuropathy caused by compression of the median nerve in the carpal tunnel. Its prevalence in the general population is between 3.8% and 5.8% [1]. CTS is caused by an ischemic injury to the median nerve caused by increased carpal tunnel internal pressure [2]. The consequences of such injuries include sensorimotor neuropathy with demyelination and structural abnormalities in the nerve. These abnormalities can reduce the neural transmission speed and action potentials of the median nerve in individuals affected by CTS [3]. Therefore, CTS is a potentially disabling condition. Hence, treatment aims to prevent and treat neurological and functional abnormalities caused by medial nerve injury [4].

For mild-to-moderate cases, conservative therapy is advocated; however, for severe cases, surgery is required [5]. Surgery may be recommended for patients with severe CTS or whose symptoms have not subsided after four to six months of conservative therapy [6].

Although postsurgical complications are infrequent, possible sequelae might have a major influence on patients’ disability, with scars, neurovascular damage, pillar discomfort, and diminished grip strength being the most common late consequences [7].

To improve neurological, functional, and aesthetic outcomes and to reduce the number of postsurgical complications in patients after surgical decompression of the carpal tunnel, numerous surgical techniques and modifications have been proposed [8,9]. Most of these studies observed differences in aesthetic effects or scar discomfort when comparing operative techniques during carpal tunnel decompression [10]. Postoperative scar discomfort can be decreased with proper wound closure. Therefore, in addition to comparing surgical approaches, some researchers examined different types of suture materials and their effect on postoperative outcomes after carpal tunnel decompression. It is unclear if absorbable sutures produce better, poorer, or similar results as non-absorbable sutures after carpal tunnel decompression [11].

Tissue adhesives have recently been used in surgical clinical practice. Tissue adhesives, such as 2-octyl cyanoacrylate (OCA), are becoming increasingly popular for strengthening wound closures because of their high tensile strength, bacteriostatic properties, and spontaneous peeling [12]. Therefore, tissue adhesives have found widespread use in a variety of surgical procedures. However, no obvious differences in dehiscence, infection, aesthetic appearance, or surgeon and patient satisfaction have been demonstrated between tissue adhesives and traditional wound-closure procedures [13].

Despite several proposed wound-closure procedures, there is a remarkable paucity of data on the potential relevance of tissue adhesives in open carpal tunnel decompression surgery. In a few comparative studies that used tissue adhesives as a material for wound closure following carpal surgery, the observed postoperative outcomes were mainly aesthetic [14]. Similarly, in our previous report, we found that cyanoacrylate-based tissue adhesion was superior in cosmetic outcomes and patient discomfort compared to conventional skin sutures for the closing of surgical wounds after CTS [15].

However, the effect of tissue adhesives as a material for wound closure following CTS decompression on medial nerve electrodiagnostic and hand function has not been investigated, despite improvements in medial nerve motor/sensory conduction and hand ability present very important endpoints of surgical decompression.

This study aimed to evaluate neurological and functional outcomes by comparing two modalities of wound closure following carpal surgery in patients who were randomly assigned to either tissue adhesive or sutures.

## Materials and methods

Patients and methods

The Plastic, Reconstructive, and Aesthetic Surgery With Burn Care Division of the University Hospital of Split in Croatia conducted a single-center, randomized, controlled, prospective, interventional, follow-up trial. As the evaluators were blinded, the study was set up as a single-blind trial. The University Hospital of Split Ethics Committee authorized the study protocol (ethics code: 500-03/22-01/41 approved) on March 31, 2022. The Consolidated Standards of Reporting Trials (CONSORT) guidelines and the Declaration of Helsinki tenets were followed in the conduct of the study. Every participant in the study gave their informed consent. This study was registered at Clinicaltrials.gov as a clinical trial (NCT05808855).

Study population

Adult patients (>18 years old) who had previously been diagnosed with CTS and were scheduled for decompression surgery in our department were included in the study. The diagnosis was based on history, physical examination (weakness of thumb abduction with thenar atrophy), and neurological examination with nerve conduction velocity assessment. The inclusion criteria for surgery included total failure of conservative therapy for more than six months and serious limitations such as thenar atrophy, thumb abduction weakness, or severe median nerve conduction impairment, as measured by electromyography. All patients had positive Tinel’s sign and Phalen’s test, which are sensitive and specific provocative tests used to diagnose CTS. During the appointment, the attending plastic surgeon made the recommendation for surgery, and the patient was booked for surgery the following month. Exclusion criteria included patients on peroral anticoagulation and/or antithrombotic therapy, previous wrist trauma or surgery on the wrist region, any other cause of neuropathy, previous allergic reactions (with lidocaine, cyanoacrylate, formaldehyde, tapes, or adhesives), a personal or family history of keloids or hypertrophic scars, and severe general illness with cachexia.

Study flow

**Figure 1:**
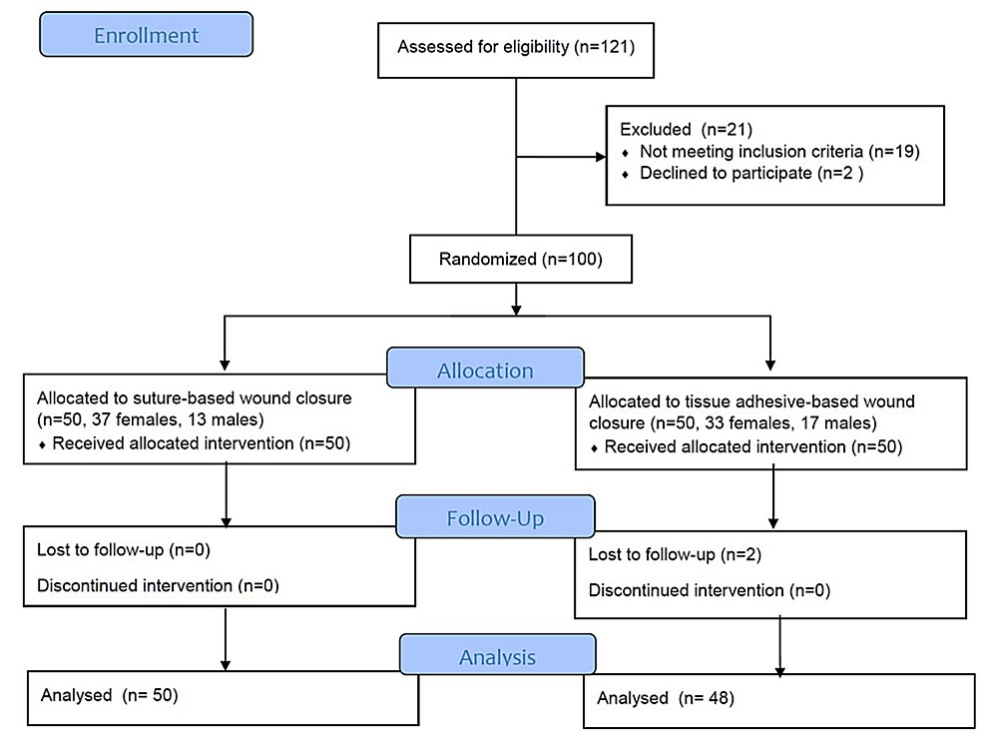
CONSORT flow diagram of the study.

Recruitment of study participants began on April 1, 2022. During the study period, 121 participants were recruited, and after applying exclusion criteria to 19 patients and two patients rejecting to participate, 100 patients were included in the trial. During the six-month follow-up in the postoperative period, two participants dropped out of the study (Figure 1). The sample size was calculated taking into account the expected variability of hand grip strength from previous studies as the main outcome measure [16], considering an alpha error of 0.05 to recognize a significant difference and 90% test power. For this study power, the minimum number of participants was estimated at 31 in each group.

Patients were randomly assigned to suture-based wound closure or tissue adhesive-based wound closure. A computer created the randomization as random numbers in a 1:1 ratio between the two interventions. The intervention was hidden from participants and plastic surgeons until they entered the operating room. Regardless of the intervention group, postoperative treatment and follow-up visits were the same. None of the patients experienced any side effects from the medication.

Procedures for interventions

Every surgical treatment was performed in the carpal tunnel and palmar soft tissues under local anesthesia with 2% lidocaine using a tourniquet. A pneumatic forearm tourniquet was used to achieve a blood-free operative field. The patients tolerated the tourniquet well, and the mean surgery decompression time was 8.71 ± 0.57 minutes. The conventional carpal canal decompression treatment involved cutting and transection of the carpal ligaments after a 15-18 mm skin incision in the radial half of the palm for all individuals [17]. Carpal tunnel decompression operations and sutures were performed by five highly experienced plastic surgeons with more than 10 years of experience in plastic surgery after obtaining a license for plastic and reconstructive surgery.

**Figure 2:**
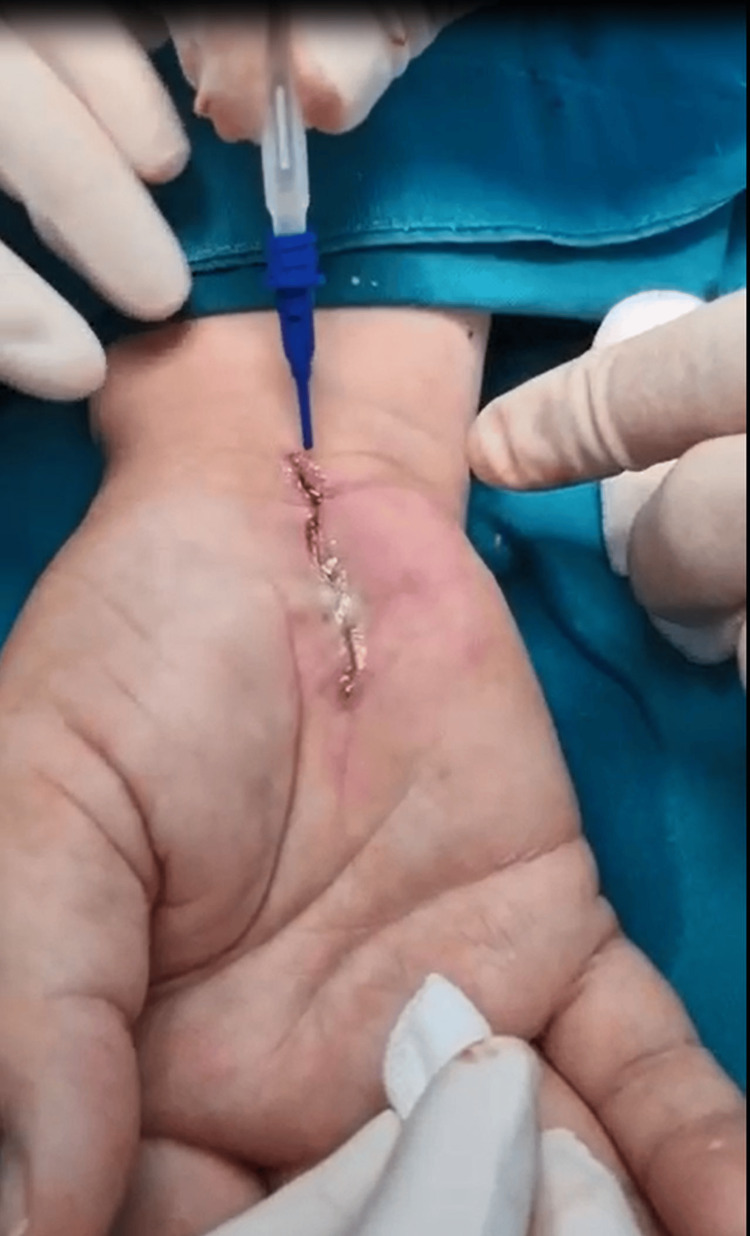
Image demonstrating wound closure with Glubran Tiss® composed of n-butyl 2 cyanoacrylate and 2-octyl cyanoacrylate.

Two wound-closure techniques were applied based on the randomization group of the patients. (1) Transcutaneous 4-0 nylon sutures (polypropylene-polyethylene monofilament, non-absorbable surgical sutures) were used to sew the skin (B. Braun Surgical, S.A. Carretera de Terrassa, Spain; Optilene® DSMP 19, 3/8 needle, thread size 4/0). (2) Following a subcutaneous buried running continuous stitch using 4-0 coated VicrylTM Plus PS-2, 3/8 (Ethicon Inc., USA), Glubran Tiss 2® (GEM S.r.l., Viareggio, Italy), a two-component skin adhesive, was applied. Glubran Tiss 2® is a synthetic surgical glue with bacteriostatic, adhesion, hemostatic, and sealing qualities that is made up of n-butyl 2-cyanoacrylate (NBCA) and OCA [18]. It instantly polymerizes into a thin, elastic film with exceptional tensile strength when applied to wet tissue, adhering firmly to the tissue’s structure. One type of bio-inert substance is polymerized glue. After each patient received 0.35 mL of Glubran Tiss® in the open wound, they relaxed for 20 seconds to allow the polymerization process to occur before bandaging (Figure 2).

Postoperative treatment included the use of a compressive bandage for one day and the administration of analgesics. If necessary, a thin plastic tube was implanted for drainage in the first two postoperative days. Daily wound dressing visits were made by the attending surgeon and nurse.

Subcutaneous buried continuous stitch was a major component of wound closure in the glue-based group, and the possible effect of an inflammatory process secondary to thread resorption needed to be estimated. Therefore, to assess the possible effect of inflammation due to suture resorption, the number of postoperative complications was recorded in both groups.

Estimation of outcome measures

Demographic data, gender, weight, height, previous illnesses, laboratory diagnostic tests, duration of symptoms, and previous physical therapy were recorded for each patient included in the study at the beginning (immediately before surgery). At the beginning of the study, hand strength was assessed with a dynamometer, and nerve conduction studies with an electroneurographic examination of the motor and sensory characteristics of the median nerve were performed on the hand planned for the operation.

Hand strength was measured with a dynamometer, and the primary result was hand grip strength. Dynamometry is a well-documented method for objectively quantifying motor outcomes and hand strength, particularly for assessing the outcome of carpal tunnel release surgery [19]. The hand grip strength test measures the motor component of all muscles involved in a hand grip and measures the motor function of maximum effort by patients who squeezed the dynamometer as hard as possible for three to five seconds. The hand grip strength of the operated arm was assessed using a hydraulic hand dynamometer, KERN MAP 130 K1 Version 1-2 (GB, Balingen, Germany). During the measurement, patients were sitting with the shoulder adducted and neutrally rotated, the elbow bent at 90°, and the forearm and wrist in a neutral position without any support. The instruction was to squeeze the handle of the dynamometer as hard as possible for three to five seconds. Subsequently, patients were exposed to median nerve conduction studies with electroneurography (ENG) to assess the function and conduction of the median nerve through the carpal tunnel (speed, latency, and amplitude of motor and sensory conduction). ENG measurements were performed with a Medelec Synergy v.11, Nicolet EDX (Domestic Natus Neurology Incorporated, WI, USA) distally across the wrist. The hands were sufficiently warmed to maintain a skin temperature of 32°C to 34°C while the patient was in a comfortable supine position. Motor outcome measures included median nerve motor latency (ms), median nerve motor amplitude (mV), and median nerve motor conduction velocity (m/s). Sensory measures included median nerve sensory conduction velocity (m/s), median nerve sensory amplitude (mV), and median nerve sensory latency (ms). The following were considered normal values for the median nerve: sensory conduction velocity >45 m/s, peak distal sensory latency <3.5 ms, sensory nerve action potential amplitude >10 mV, distal motor latency <4.2 ms, distal motor amplitude >5 mV, and motor conduction velocity >50 m/s [20]. After sensory and motor ENG measurements, the total ENG Bland score was calculated for each subject [21]. The Bland scale ranges from 0 to 6, where 0 represents a normal finding, and 6 represents the worst finding with sensory and motor potentials effectively unrecordable.

At the beginning of the study, the Boston Carpal Tunnel Questionnaire (BCTQ) was administered to every participant. BCTQ [22] was completed by the patient and consists of two scales: the Boston Symptom Severity Scale (SSS) and the Functional Status Scale (FSS). The Boston SSS uses a five-point rating scale for 11 questions on pain, paresthesia, numbness, weakness, nocturnal symptoms, and difficulty grasping, with a final score ranging from 11 to 55. A higher score suggests a higher level of impairment. The FSS also uses a five-point rating scale for eight questions to estimate the degree of difficulty in typical daily activities (writing, buttoning clothes, holding a book while reading, gripping a telephone handle, opening jars, performing household chores, carrying grocery bags, bathing, and dressing). The final score ranges from 8 to 40. A higher score indicates a greater degree of disability. After six months, hand grip strength measurements, BCTQ scores, and measurements of sensory and motor neuroelectrical conduction properties of the median nerve with Bland scores were again determined for all included participants. Changes (delta) in all variables compared to the initial values were calculated.

Statistical analysis

Calculations and data for descriptive statistics were expressed as the median with interquartile range if the data were non-normally distributed, or as the arithmetic mean ± standard deviation if the data were normally distributed. The Kolmogorov-Smirnov test was employed to estimate the distributional normality of quantitative variables. To compare qualitative data between groups, Fisher’s exact test and chi-square test were used. A paired and an unpaired Student’s t-test were used to compare quantitative data. To analyze and compare quantitative variables with a non-normal distribution, the Mann-Whitney test for unpaired variables and the Wilcoxon signed ranks test for paired variables were used. The statistical analysis was performed using SPSS Statistics for Windows version 26.0 (IBM Corp., Armonk, NY, USA). Two-tailed p-values less than 0.05 were considered significant.

## Results

The study participants consisted of 100 patients (30 males and 70 females) randomly assigned in a 1:1 ratio for wound closure with glue (n = 50) and sutures (n = 50). After postoperative follow-up, 98 participants were assessed. The age of the entire cohort was 61.56 ± 12.03 years. The mean surgery decompression time was 8.71 ± 0.57 minutes. We could not find differences between surgery decompression time between the glue-based and suture-based groups (8.74 ± 0.57 vs. 8.67 ± 0.57 minutes, p = 0.264). The right-side surgery was performed on 57 patients, while the left-side surgery was performed on 43 patients.

To measure the possible effect of inflammation due to suture resorption in the glue plus subcutaneous suture-based wound closure group, early and late postoperative complications were compared between both groups. In the entire cohort, 12 individuals experienced the following early postoperative complications after 15 days: redness (five cases), dehiscence (two cases), hemorrhage (two cases), infection (one case), allergic dermatitis (one case), and secretion (one case). Following a 12-week surgical period, which is the time after thread resorption in the group with subcutaneous continuous sutures, eight patients experienced late complications, five had granulomas, two had secretions, and one had an infection. There were no statistically significant differences in the number of early or late complications between the glue plus subcutaneous suture-based wound closure and the transcutaneous suture-based wound closure groups (χ^2^ < 0.001, p = 0.620, and χ^2^ = 0.54, p = 0.375, respectively).

Differences in functional, clinical, and electroneurographic parameters before and after decompression surgery for all participants and the groups are presented in Tables 1-3. From these data, it is evident that a vast majority of parameters significantly improved after surgical decompression of the carpal tunnel, with small exceptions between the groups. In the glue-based wound closure group, the difference in motor amplitude of the median nerve before and after decompression surgery did not reach a significant difference. The difference in motor velocity of the median nerve before and after decompression surgery was not significant in the suture-based wound closure group.

**Table 1:** Differences in hand grip strength, clinical features, and electroneurographic conduction parameters of the median nerve before and after decompression surgery for all participants (N = 98) (Student’s t-test for dependent samples or Wilcoxon signed-rank test). P-values for significant differences (two-tailed) are bolded. Data are presented as the arithmetic mean ± standard deviation, or median (with interquartile range) for non-parametric data. BCTQ = Boston Carpal Tunnel Questionnaire; SSS = symptom severity scale; FSS = functional status scale

	Before surgery	6 months after surgery	
	Mean ± SD	Mean ± SD	P-values
Hand grip strength (kg)	18.52 ± 5.64	23.20 ± 6.40	<0.001
Sensory latency (ms)	3.55 ± 1.59	2.72 ± 0.99	<0.001
Sensory amplitude (mV)	4.55 ± 3.76	8.78 ± 5.58	<0.001
Sensory velocity (m/s)	29.33 ± 15.52	38.65 ± 14.40	<0.001
Motor latency (ms)	5.54 ± 1.60	4.46 ± 1.40	<0.001
Motor amplitude (mV)	6.55 ± 4.17	7.70 ± 3.81	0.015
Motor velocity (m/s)	50.29 ± 12.61	55.14 ± 8.10	0.001
BCTQ-SSS	41.95 ± 8.06	22.45 ± 6.34	<0.001
BCTQ-FSS	27.66 ± 9.17	14.46 ± 5.09	<0.001
Bland score	4.00 (3.00-5.00)	3.00 (3.00-3.00)	<0.001

**Table 2:** Differences in hand grip strength, clinical features, and electroneurographic conduction parameters of the median nerve before and after decompression surgery for glue-based wound closure participants (N = 48) (Student’s t-test for dependent samples or Wilcoxon-signed rank test). P-values for significant differences (two-tailed) are bolded. Data are presented as the arithmetic mean ± standard deviation, or median (with interquartile range) for non-parametric data. BCTQ = Boston Carpal Tunnel Questionnaire; SSS = symptom severity scale; FSS = functional status scale

	Before surgery	6 months after surgery	
	Mean ± SD	Mean ± SD	P-values
Hand grip strength (kg)	19.48 ± 5.95	25.06 ± 6.69	<0.001
Sensory latency (ms)	3.73 ± 1.90	2.65 ± 0.60	<0.001
Sensory amplitude (mV)	5.01 ± 4.00	10.08 ± 5.50	<0.001
Sensory velocity (m/s)	27.73 ± 15.24	42.22 ± 11.04	<0.001
Motor latency (ms)	5.44 ± 1.61	4.39 ± 1.82	0.002
Motor amplitude (mV)	7.28 ± 3.91	8.08 ± 3.80	0.241
Motor velocity (m/s)	51.62 ± 8.11	56.38 ± 5.04	<0.001
BCTQ-SSS	42.65 ± 9.10	23.19 ± 6.41	<0.001
BCTQ-FSS	26.81 ± 6.62	14.08 ± 5.61	<0.001
Bland score	3.00 (3.00-4.75)	3.00 (3.00-3.00)	0.002

**Table 3:** Differences in hand grip strength, clinical features, and electroneurographic conduction parameters of the median nerve before and after decompression surgery for suture-based wound closure participants (N = 50) (Student’s t-test for dependent samples or Wilcoxon signed-rank test). P-values for significant differences (two-tailed) are bolded. Data are presented as the arithmetic mean ± standard deviation, or median (with interquartile range) for non-parametric data. BCTQ = Boston Carpal Tunnel Questionnaire; SSS = symptom severity scale; FSS = functional status scale

	Before surgery	6 months after surgery	
	Mean ± SD	Mean ± SD	P-value
Hand grip strength (kg)	17.61 ± 5.22	21.41 ± 5.62	<0.001
Sensory latency (ms)	3.35 ± 1.14	2.80 ± 1.30	0.009
Sensory amplitude (mV)	4.10 ± 3.49	7.54 ± 5.41	<0.001
Sensory velocity (m/s)	30.86 ± 15.79	35.23 ± 16.40	0.015
Motor latency (ms)	5.64 ± 1.60	4.53 ± 0.85	<0.001
Motor amplitude (mV)	5.86 ± 4.33	7.34 ± 3.82	0.024
Motor velocity (m/s)	49.04 ± 15.70	53.98 ± 10.08	0.055
BCTQ-SSS	41.28 ± 6.95	21.74 ± 6.27	<0.001
BCTQ-FSS	28.48 ± 11.09	14.82 ± 4.57	<0.001
Bland score	4.00 (3.00-5.00)	3.00 (3.00-3.00)	0.001

**Figure 3:**
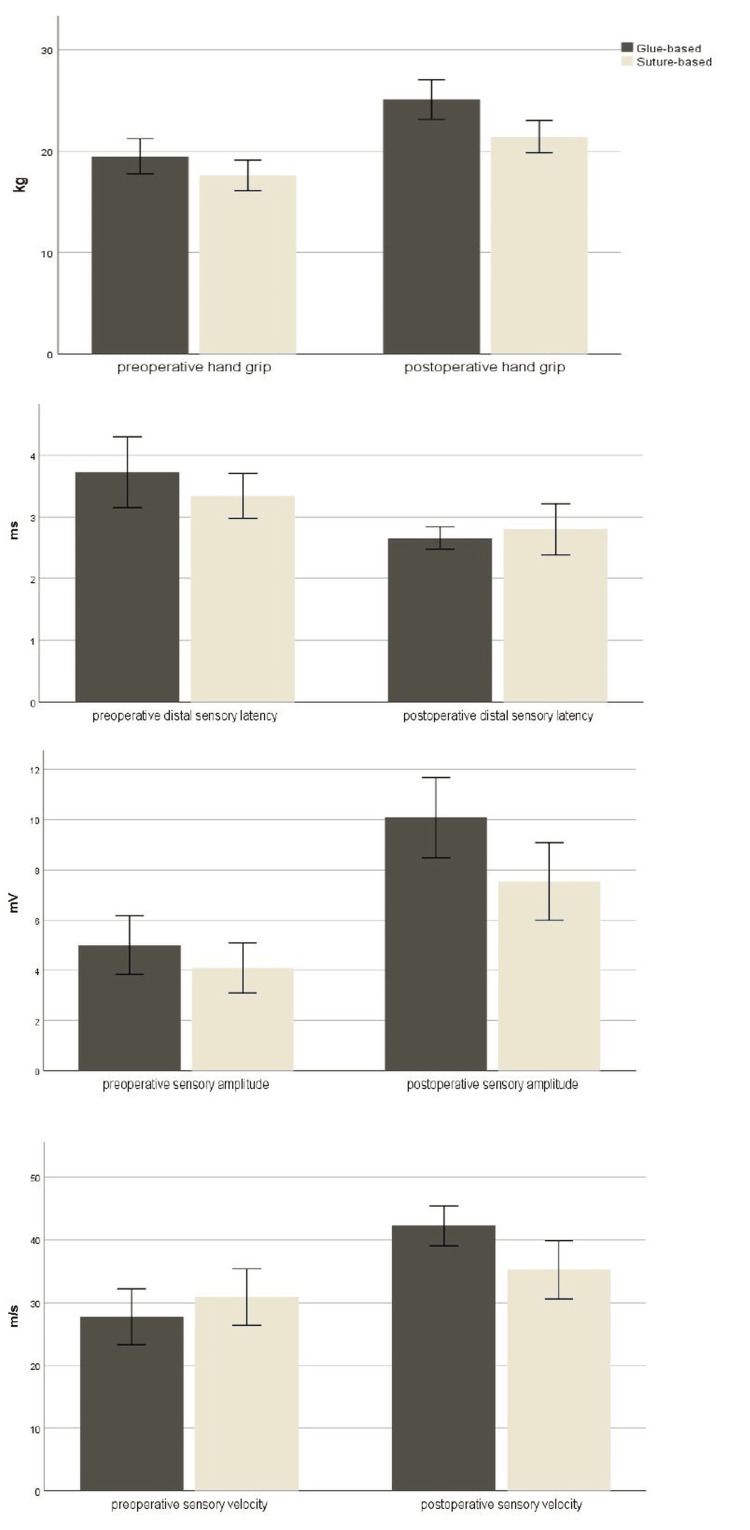
The differences between glue-based and suture-based wound-closure techniques in preoperative and postoperative values of hand grip, distal sensory latency, sensory action potential amplitude, and sensory conduction velocity of the median nerve.

Differences in functional, clinical, and electroneurographic parameters between glue-based wound closure and suture-based wound closure patients are demonstrated in Table 4. Significant differences between the two wound-closure techniques were found in postoperative hand grip strength, delta pre- and postsurgical sensory latency, postoperative sensory amplitude, and postoperative sensory velocity; these results favored glue-based wound closure. These significant differences between glue-based and suture-based wound-closure techniques with preoperative and postoperative values are shown in Figure 3.

**Table 4:** Differences in hand grip strength, clinical features, and electroneurographic conduction parameters of the median nerve between glue-based wound closure and suture-based wound closure (Student’s t-test for independent samples or Mann-Whitney test for non-parametric data). P-values for significant differences are bolded. Data are presented as the arithmetic mean ± standard deviation or median (with interquartile range) for non-parametric data. BCTQ = Boston Carpal Tunnel Questionnaire; SSS = symptom severity scale; FSS = functional status scale

	Glue-based technique	Suture-based technique	
	N = 48	N = 50	P-value
	Mean ± SD	Mean ± SD	
Age (years)	63.02 ± 12.967	60.10 ± 10.95	0.113
Body mass index (kg/m^2^)	24.79 ± 3.172	25.04 ± 2.25	0.325
Hand grip strength, preoperative (kg)	19.48 ± 5.95	17.61 ± 5.22	0.051
Hand grip strength, 6 months (kg)	25.06 ± 6.69	21.41 ± 5.62	0.002
Delta, hand grip strength (post-pre) (kg)	5.58 ± 3.29	3.80 ± 2.50	0.002
Sensory latency, preoperative (ms)	3.73 ± 1.90	3.35 ± 1.14	0.137
Sensory latency, 6 months (ms)	2.69 ± 0.60	3.14 ± 1.96	0.067
Delta, sensory latency (pre-post) (ms)	1.07 ± 1.61	0.54 ± 1.25	0.049
Sensory amplitude, preoperative (mV)	5.01 ± 4.00	4.10 ± 3.49	0.117
Sensory amplitude, 6 months (mV)	10.08 ± 5.50	7.54 ± 5.41	0.012
Delta, sensory amplitude (post-pre) (mV)	5.07 ± 4.39	3.43 ± 3.95	0.028
Sensory velocity, preoperative (m/s)	27.73 ± 15.24	30.86 ± 15.79	0.161
Sensory velocity, 6 months (m/s)	42.22 ± 11.04	35.23 ± 16.40	0.008
Delta, sensory velocity (post-pre) (m/s)	14.48 ± 13.25	4.38 ± 12.33	<0.001
Motor latency, preoperative (ms)	5.44 ± 1.61	5.64 ± 1.60	0.273
Motor latency, 6 months (ms)	4.39 ± 1.82	4.53 ± 0.85	0.310
Delta, motor latency (pre-post) (ms)	1.06 ± 2.24	1.11 ± 1.69	0.446
Motor amplitude, preoperative (mV)	7.28 ± 3.91	5.86 ± 4.33	0.046
Motor amplitude, 6 months (mV)	8.08 ± 3.80	7.34 ± 3.82	0.170
Delta, motor amplitude (post-pre) (mV)	0.80 ± 4.69	1.48 ± 4.50	0.233
Motor velocity, preoperative (m/s)	51.62 ± 8.11	49.04 ± 15.70	0.158
Motor velocity, 6 months (m/s)	56.38 ± 5.04	53.98 ± 10.08	0.073
Delta, motor velocity (post-pre) (m/s)	4.76 ± 8.56	4.94 ± 17.77	0.474
BCTQ-SSS, preoperative	42.65 ± 9.10	41.28 ± 6.95	0.202
BCTQ-SSS, 6 months	23.19 ± 6.41	21.74 ± 6.27	0.130
Delta, BCTQ-SSS (post-pre)	19.46 ± 7.51	19.54 ± 6.03	0.476
BCTQ-FSS, preoperative	26.81 ± 6.62	28.48 ± 11.09	0.185
BCTQ-FSS, 6 months	14.08 ± 5.61	14.82 ± 4.57	0.238
Delta, BCTQ-FSS (post-pre)	12.73 ± 5.97	13.66 ± 9.50	0.282
Bland score, preoperative	3.00 (3.00-4.75)	4.00 (3.00-5.00)	0.142
Bland score, 6 months	3.00 (3.00-3.00)	3.00 (3.00-3.00)	0.245
Delta, Bland score (pre-post)	0.00 (0.00-1.00)	0.50 (0.00-1.00)	0.224

In the glue-based wound closure group, 23 (47.9%) patients achieved a postoperative sensory velocity greater than 45 m/s, while in the suture group, only 11 (22.0%) patients achieved a postoperative sensory velocity greater than 45 m/s. The difference was statistically significant (χ^2^ = 7.260; p = 0.006). A greater number of participants in the glue-based wound closure group achieved an increase in sensory velocity after surgery compared to the suture group (44 or 91.8% vs. 38 or 76.0%), and the difference was statistically significant (χ^2^ = 4.400; p = 0.033). In the glue-based wound closure group, 43 (89.6%) patients achieved postoperative peak distal sensory latency of less than 3.5 ms compared to 42 (84.0% %) in the suture group. The difference did not reach significance (χ^2^ = 0.664; p = 0.304). In the glue-based wound closure group, 19 (39.6%) patients reached a sensory nerve action potential amplitude higher than 10 mV compared to 14 (28.0%) in the suture group. The difference did not reach significance (χ^2^ = 1.471; p = 0.159). In the glue-based wound closure group, 29 (60.42%) patients achieved postoperative motor latency of less than 4.2 ms compared to only 19 (38.00%) in the suture group. The difference was statistically significant (χ^2^ = 4.925; p = 0.022). In the glue-based wound closure group, there were more patients with postoperative motor amplitude greater than 5 mV than in the suture group (40 patients, or 83.3%, vs. 37 patients, or 74.0%), but statistical significance was not reached (χ^2^ = 1.267; p = 0.190). The glue-based wound closure group had 44 (93.6%) patients with a postoperative motor velocity greater than 50 m/s compared to 42 (84.0%) in the suture group, and the difference was not significant (χ^2^ = 2.229; p = 0.120).

## Discussion

The results of this randomized clinical study demonstrated significant six-month postoperative augmentation of hand grip as an important functional indicator, as well as an increase in median nerve conduction parameters in the group of patients whose surgical wound was closed with tissue glue and subcutaneous sutures compared to the group whose surgical wound was closed with transcutaneous sutures. We were unable to demonstrate differences in the BCTQ between the two surgical wound-closure techniques. However, electrophysiologic indices estimated by median nerve conduction parameters are important markers of CTS severity and have been found to be correlated with hand functional disability levels. For example, nerve conduction measured as median motor latency >4.2 ms and median sensory latency >3.5 ms are considered markers of severe CTS [3]. In our study, we showed a significant postoperative improvement in sensory median nerve conduction parameters in the group of patients whose surgical wound was closed with tissue glue. Furthermore, in the glue-based wound closure group, significantly more patients achieved a postoperative sensory velocity >45 m/s, a postoperative peak distal sensory latency <3.5 ms, and a postoperative motor latency <4.2 ms. Likewise, we showed significantly better postoperative hand grip results in the group of patients whose wound was closed with tissue glue, which we consider an important result. Hence, the dynamometric measurement of grip strength represents a major postoperative outcome measure and has an impact on the long-term outcomes of patients with CTS [19].

The results of our study also showed a statistically significant postoperative improvement in electroneurographic, clinical, and functional parameters in all patients, as well as in separate groups of patients. This is similar to the results of previous studies, which also showed a statistically significant improvement in the electrophysiological parameters of the median nerve, as well as an improvement in the clinical characteristics of the hand, including Boston questionnaire results, after decompression surgery for CTS [23,24]. This proves the effectiveness of surgical treatment for CTS compared to other treatment modalities. For example, in a study of 18 CTS patients, El-Hajj et al. [25] found an 82.3% improvement in nerve conduction parameters after surgery, which increased to 88.2% after nine months.

Therefore, the measurement of the electroneurographic parameters of the median nerve with or without measurement of hand functional capabilities serves as an important outcome to compare the effectiveness of various treatment modalities [26]. A randomized clinical trial comparing local corticosteroid injection and decompressive surgery for CTS found significant improvements in distal motor latency, sensory conduction velocity, and sensory amplitude in the surgery group 12 months after treatment [27].

Likewise, these electrophysiological neurological outcomes as well as functional outcomes serve to evaluate the success of a surgical CTS treatment [28]. Accordingly, these electrophysiological and/or functional outcomes were used to evaluate differences between standard surgical procedures and surgical modifications, such as mini-invasive techniques like endoscopic and mini-open approaches to the carpal tunnel [29]. In 62 patients with CTS, Tian et al. [30] reported no significant differences between open or endoscopic carpal tunnel release in postoperative improvements of clinical and electromyography parameters tested before the operation and three months after the operation. In one study, postoperative median nerve distal motor latency, motor transmission velocity, and sensory nerve action potential were measured three, six, and 12 months after surgery and did not differ between the groups following CTS surgery. The control group underwent standard surgical decompression, and the intervention group underwent surgical decompression with a single injection of platelet-rich plasma introduced through the mini-vacuum drain [31]. Similarly, modified Camitz opponensplasty did not correlate with neurologic and muscle recovery in a study of 21 participants with severe CTS [32]. A retrospective study that compared endoscopic carpal tunnel release with open carpal tunnel release did not find any differences in outcomes, including electromyographic abnormalities [33]. A similar prospective study compared endoscopic carpal tunnel decompression with open carpal tunnel decompression and did not find differences between the two techniques in functional and electromyography outcomes, despite a significant shortening in median nerve motor distal latency and an increase in the velocity of sensory conductions in both groups [34]. A retrospective analysis of 114 CTS patients compared two surgical procedures, namely, classical open neurolysis of the median nerve with flexor retinaculum lengthening according to the Simonetta technique, and demonstrated that ligamentoplasty according to the Simonetta technique led to better manual force in 10 years of follow-up [35]. A prospective, randomized, double-blind, controlled trial involving 50 CTS patients compared the longitudinal epineurotomy of the median nerve with the simple dissection of the carpal ligament and found no differences in distal motor latency, distal sensory latency, or grip strength after 180 days of follow-up [36]. Boumediane et al. [37], in 31 patients with recurrent CTS, compared two modifications of surgical treatments, including the application of carboxymethylcellulose/polyethylene oxide gel added to the Canaletto® implant with such anti-adhesion gel alone. The study could not demonstrate postoperative differences in the distal motor latency and sensory nerve conduction speed between the groups. Bilge et al [38]. compared 200 CTS patients divided after mini-incision open surgery into two groups, i.e., whether they received or did not receive local administration of anti-adhesion gel consisting of hyaluronic acid-carboxymethylcellulose. They could not demonstrate a significant difference between groups in the Boston questionnaire after the operation. In conclusion, the majority of previous studies comparing different surgical techniques found no significant differences in functional and/or neurological outcomes between surgical modalities.

In recent years, cyanoacrylate-based skin glue has grown in popularity in various surgical procedures for ensuring and maintaining wound closure due to the acknowledged benefits of these glues in their ability to physically isolate the surgical incision and reduce the risk of surgical site infection [39,40]. Although skin adhesives have been extensively explored for wound closure in various surgical procedures, only a few studies have assessed skin adhesives following carpal tunnel decompression and reported only aesthetic outcomes. A prospective randomized controlled trial compared adhesive tape and a tissue adhesive (OCA) applied after primary closure with a 4-0 absorbable suture to different halves of the same surgical incision after carpal tunnel decompression and reported that the adhesive strips provided significant improvement in cosmetic outcomes observed by a surgeon three months after the surgery [14]. In a prospective randomized study, Sinha et al. [41] compared the closure of hand surgery wounds (including 22 CTS patients) with a tissue adhesive (NBCA) or with the standard wound-closure technique (4-0 monofilament suture) and could not demonstrate significant differences in the cosmetic outcomes at two and six weeks after surgery. Finally, we previously reported in a randomized controlled trial that wound closure using cyanoacrylate-based adhesion material compared to a control group that used sutures as a wound-closure procedure following open CTS was significantly better in terms of aesthetic outcomes, discomfort, and patient compliance at two and six weeks postoperatively [15]. This randomized clinical trial is the first to demonstrate the differences between wound closure with tissue adhesives and classical wound closure with sutures in patients with CTS concerning neurological and functional outcomes. However, it should be emphasized that these significant postoperative differences between the groups in hand strength and median nerve electroneurographic parameters improvement are exclusively the result of the differences in the two surgical techniques for closing the surgical wound, i.e., the one that uses glue plus subcutaneous running continuous stitch and the one that uses transcutaneous sutures. The same experienced operators operated both groups of patients with no difference in the duration of the procedure. Likewise, the influence of inflammation secondary to thread resorption in the glue subcutaneous stitch group probably did not affect the outcome measures because no significant difference was shown between the postoperative complications in the two groups. Finally, the obtained significant postoperative differences in outcome measures can not only be attributed exclusively to the use of tissue glue but also to the difference between subcutaneous and transcutaneous sutures. Specifically, patients with transcutaneous sutures are spared the discomfort and pain of suture removal. Furthermore, classic transcutaneous suturing involves a larger area of the sutured tissue (skin and subcutaneous tissue). In contrast, with a subcutaneous suture using tissue glue, the tension of the wound is equally distributed with reduced pressure of the sutures on the skin where the nociceptors are located, and, therefore, this group can start using the operated hand earlier.

Limitations

This study has certain limitations. First, this trial was restricted to a single center. Second, the six-month follow-up period is not long enough to conclude the long-term effects of the intervention. Third, to further understand the impact of the intervention, it is necessary to monitor additional clinical, histological, and biomechanical parameters. Fourth, the obtained differences in outcome measures are not exclusively a consequence of the application of tissue glue but also of the use of a continuous subcutaneous suture, which can significantly affect the strength and tension of the wound closure.

## Conclusions

This randomized controlled trial demonstrated that cyanoacrylate-based adhesion material in combination with a subcutaneous continuous stitch for wound closure after open CTS decompression has an advantage over transcutaneous sutures in terms of functional markers measured as hand grip strength and neurological indices measured as median sensory nerve conduction parameters. Wound closure after CTS decompression surgery can be efficiently and safely realized with cyanoacrylate-based tissue adhesives, with anticipated beneficial effects on median nerve recovery and the improvement of hand strength. Larger multicenter trials with longer follow-ups and additional outcomes are required to further strengthen the conclusions of this study.

## References

[1] Tekin F, Sürmeli M, Şimşek H, Ceran C, Tezcan S, Taner ÖF, Şimşek G (2015). Comparison of the histopathological findings of patients with diabetic and idiopathic carpal tunnel syndrome. Int Orthop.

[2] de Krom MC, de Krom CJ, Spaans F (2009). [Carpal tunnel syndrome: diagnosis, treatment, prevention and its relevance to dentistry]. Ned Tijdschr Tandheelkd.

[3] Daliri B O M, Azhari A, Khaki S, Hajebi Khaniki S, Moradi A (2022). Which psychological and electrodiagnostic factors are associated with limb disability in patients with carpal tunnel syndrome?. Clin Orthop Relat Res.

[4] Thomsen NO, Rosén I, Dahlin LB (2010). Neurophysiologic recovery after carpal tunnel release in diabetic patients. Clin Neurophysiol.

[5] Jiménez Del Barrio S, Bueno Gracia E, Hidalgo García C, Estébanez de Miguel E, Tricás Moreno JM, Rodríguez Marco S, Ceballos Laita L (2018). Conservative treatment in patients with mild to moderate carpal tunnel syndrome: a systematic review. Neurologia (Engl Ed).

[6] Wipperman J, Goerl K (2016). Carpal tunnel syndrome: diagnosis and management. Am Fam Physician.

[7] Kluge W, Simpson RG, Nicol AC (1996). Late complications after open carpal tunnel decompression. J Hand Surg Br.

[8] Bolster M, Schipper C, Van Sterkenburg S, Ruettermann M, Reijnen M (2013). Single interrupted sutures compared with Donati sutures after open carpal tunnel release: a prospective randomised trial. J Plast Surg Hand Surg.

[9] Suwannaphisit S, Aonsong W, Suwanno P, Yuenyongviwat V (2021). Comparing the running subcuticular technique versus the Donati technique in open carpal tunnel release: a randomized controlled trial. J Orthop Surg Res.

[10] Ahcan U, Arnez ZM, Bajrović F, Zorman P (2002). Surgical technique to reduce scar discomfort after carpal tunnel surgery. J Hand Surg Am.

[11] Wade RG, Wormald JC, Figus A (2018). Absorbable versus non-absorbable sutures for skin closure after carpal tunnel decompression surgery. Cochrane Database Syst Rev.

[12] Coulthard P, Esposito M, Worthington HV, van der Elst M, van Waes OJ, Darcey J (2010). Tissue adhesives for closure of surgical incisions. Cochrane Database Syst Rev.

[13] Dumville JC, Coulthard P, Worthington HV (2014). Tissue adhesives for closure of surgical incisions. Cochrane Database Syst Rev.

[14] McQuillan TJ, Vora M, Hawkins J, Kenney D, Diaz R, Ladd AL (2022). Adhesive taping shows better cosmetic outcomes than tissue adhesives for sutured upper extremity incisions: a single-blind prospective randomized controlled trial. Orthopedics.

[15] Sunjic Roguljic V, Roguljic L, Kovacic V, Jukic I (2023). A comparison of tissue adhesive material and suture as wound-closure techniques following carpal tunnel decompression: a single-center randomized control trial. J Clin Med.

[16] Fernandes CH, Meirelles LM, Santos JB, Fernandes M, Nakachima LR, Faloppa F (2023). An intraindividual comparison of open versus Paine retinaculotome release for bilateral carpal tunnel syndrome. Rev Bras Ortop (Sao Paulo).

[17] Steinberg DR (2002). Surgical release of the carpal tunnel. Hand Clin.

[18] Kakaei F, Seyyed Sadeghi MS, Sanei B, Hashemzadeh S, Habibzadeh A (2013). A randomized clinical trial comparing the effect of different haemostatic agents for haemostasis of the liver after hepatic resection. HPB Surg.

[19] Fernandes CH, Meirelles LM, Raduan Neto J, Nakachima LR, Dos Santos JB, Faloppa F (2013). Carpal tunnel syndrome with thenar atrophy: evaluation of the pinch and grip strength in patients undergoing surgical treatment. Hand (N Y).

[20] Faour-Martín O, Martín-Ferrero MA, Almaraz-Gómez A, Vega-Castrillo A (2012). The long-term post-operative electromyographic evaluation of patients who have undergone carpal tunnel decompression. J Bone Joint Surg Br.

[21] Bland JD (2000). A neurophysiological grading scale for carpal tunnel syndrome. Muscle Nerve.

[22] Levine DW, Simmons BP, Koris MJ, Daltroy LH, Hohl GG, Fossel AH, Katz JN (1993). A self-administered questionnaire for the assessment of severity of symptoms and functional status in carpal tunnel syndrome. J Bone Joint Surg Am.

[23] Bulut T, Sener U, Yağdi S, Kazimoğlu C, Sener M (2011). Relationship between clinical and electrophysiological results in surgically treated carpal tunnel syndrome. Eklem Hastalik Cerrahisi.

[24] Yilmaz N, Akdemir G, Gezici AR (2010). Electrophysiological and clinical assessment of response to surgery in carpal tunnel. Int J Neurosci.

[25] El-Hajj T, Tohme R, Sawaya R (2010). Changes in electrophysiological parameters after surgery for the carpal tunnel syndrome. J Clin Neurophysiol.

[26] Deng X, Chau LP, Chiu SY, Leung KP, Hu Y, Ip WY (2019). Screening of axonal degeneration in carpal tunnel syndrome using ultrasonography and nerve conduction studies. J Vis Exp.

[27] Andreu JL, Ly-Pen D, Millán I, de Blas G, Sánchez-Olaso A (2014). Local injection versus surgery in carpal tunnel syndrome: neurophysiologic outcomes of a randomized clinical trial. Clin Neurophysiol.

[28] Pourmokhtari M, Mazrooyi M, Vosoughi AR (2021). Conservative or surgical treatment of carpal tunnel syndrome based on the severity and patient risk factors. Musculoskelet Surg.

[29] Chammas M (2014). Carpal tunnel syndrome. Chir Main.

[30] Tian Y, Zhao H, Wang T (2007). Prospective comparison of endoscopic and open surgical methods for carpal tunnel syndrome. Chin Med Sci J.

[31] Yasak T, Özkaya Ö, Ergan Şahin A, Çolak Ö (2022). Electromyographic and clinical investigation of the effect of platelet-rich plasma on peripheral nerve regeneration in patients with diabetes after surgery for carpal tunnel syndrome. Arch Plast Surg.

[32] Durban CM, Antolin B, Sau CY, Li SW, Ip WY (2017). Thumb function and electromyography result after modified Camitz tendon transfer. J Hand Surg Asian Pac Vol.

[33] Calotta NA, Lopez J, Deune EG (2017). Improved surgical outcomes with endoscopic carpal tunnel release in patients with severe median neuropathy. Hand (N Y).

[34] Gümüştaş SA, Ekmekçi B, Tosun HB, Orak MM, Bekler Hİ (2015). Similar effectiveness of the open versus endoscopic technique for carpal tunnel syndrome: a prospective randomized trial. Eur J Orthop Surg Traumatol.

[35] Faour-Martín O, Martín-Ferrero MÁ, Vega Castrillo A, Almaraz-Gómez A, Valverde-García JA, Amigo Liñares L, Red-Gallego MÁ (2013). Long-term effects of preserving or splitting the carpal ligament in carpal tunnel operation. J Plast Surg Hand Surg.

[36] Crnković T, Bilić R, Trkulja V, Cesarik M, Gotovac N, Kolundžić R (2012). The effect of epineurotomy on the median nerve volume after the carpal tunnel release: a prospective randomised double-blind controlled trial. Int Orthop.

[37] Boumediane M, Meyer N, Facca S, Pizza C, Liverneaux P (2021). Revision surgery for carpal tunnel syndrome: a retrospective study comparing the combination of Canaletto® and Dynavisc® gel versus Dynavisc® gel alone. Hand Surg Rehabil.

[38] Bilge A, Ulusoy RG, Ozturk O, Ozturk IA, Aykut S (2017). Carpal tunnel syndrome surgery anti-adhesion gel is effective?. Acta Chir Orthop Traumatol Cech.

[39] Rushbrook JL, White G, Kidger L, Marsh P, Taggart TF (2014). The antibacterial effect of 2-octyl cyanoacrylate (Dermabond®) skin adhesive. J Infect Prev.

[40] Lee CS, Han SR, Kye BH (2020). Surgical skin adhesive bond is safe and feasible wound closure method to reduce surgical site infection following minimally invasive colorectal cancer surgery. Ann Surg Treat Res.

[41] Sinha S, Naik M, Wright V, Timmons J, Campbell AC (2001). A single blind, prospective, randomized trial comparing n-butyl 2-cyanoacrylate tissue adhesive (Indermil) and sutures for skin closure in hand surgery. J Hand Surg Br.

